# c-MET Overexpression Drives AKT Activation, and Combined Inhibition Synergistically Enhances Therapeutic Sensitivity in Non-Small-Cell Lung Cancer

**DOI:** 10.3390/cells15131155

**Published:** 2026-06-25

**Authors:** Pratheesh Kumar Poyil, Rafia Begum, Saravanan Thangavel, Khadija Al-Obaisi, Abdul K. Siraj

**Affiliations:** 1 Experimental Therapeutics, Research & Innovation, King Faisal Specialist Hospital and Research Center, Riyadh 11211, Saudi Arabia; brafia@kfshrc.edu.sa; 2 Genomic Analytics, Research & Innovation, King Faisal Specialist Hospital and Research Centre, Riyadh 11211, Saudi Arabia; tsaravanan97@kfshrc.edu.sa; 3 Translational Oncology Research, Research & Innovation, King Faisal Specialist Hospital and Research Center, Riyadh 11211, Saudi Arabia; kalobaisi@kfshrc.edu.sa (K.A.-O.); asiraj@kfshrc.edu.sa (A.K.S.)

**Keywords:** c-MET, AKT signaling, non-small-cell lung cancer (NSCLC), apoptosis, combination therapy

## Abstract

**Highlights:**

**What are the main findings?**

- c-MET overexpression is associated with AKT activation and poor clinical outcome in NSCLC.
- Combined inhibition of c-MET and AKT synergistically suppresses tumor growth and enhances apoptosis in NSCLC models in vitro and in vivo.

**What are the implications of the main findings?**

- AKT functions as a critical downstream survival pathway in c-MET-driven NSCLC and may contribute to resistance against c-MET-targeted therapy.
- Dual targeting of c-MET and AKT represents a promising therapeutic strategy for improving treatment efficacy in NSCLC.

**Abstract:**

Aberrant activation of c-MET signaling contributes to tumor progression and resistance to therapy in non-small-cell lung cancer (NSCLC), yet its therapeutic significance remains incompletely understood. In this study, we evaluated c-MET expression and its association with AKT activation and clinical outcomes using a tissue microarray cohort and publicly available datasets. c-MET overexpression was significantly associated with increased p-AKT expression and showed a trend toward poorer overall survival in the tissue microarray cohort, while analysis of the TCGA LUAD dataset confirmed a significant association with reduced survival (log-rank *p* = 0.0223; HR = 1.234, 95% CI: 1.029–1.480). Functional studies demonstrated that pharmacological inhibition of c-MET suppressed cell proliferation and induced caspase-dependent mitochondrial apoptosis in NSCLC cell lines. Mechanistically, c-MET inhibition resulted in AKT inactivation, identifying AKT as a key downstream mediator of c-MET signaling. Notably, combined inhibition of c-MET (PHA665752) and AKT (MK2206) exhibited strong synergistic effects, significantly enhancing apoptosis and reducing cell viability compared to single-agent treatments. These findings were further validated in vivo, where combination therapy markedly delayed tumor growth without significant toxicity. Collectively, our results highlight c-MET-driven AKT activation as a key oncogenic mechanism and support dual c-MET/AKT targeting as a promising therapeutic strategy for NSCLC.

## 1. Introduction

Lung cancer remains a significant global health challenge and the leading cause of cancer mortality, primarily due to advanced-stage diagnosis, marked molecular complexity, and the acquisition of resistance to anticancer therapies [1,2]. Despite advances in targeted therapies and immunotherapy, a substantial proportion of patients either fail to respond or develop disease progression, thereby reinforcing the need to identify additional targetable molecular pathways and develop mechanism-based combination therapies [2,3].

The HGF/c-MET signaling axis regulates multiple physiological processes, including cellular growth, survival, migration, and tissue regeneration [4]. Aberrant activation of c-MET, through overexpression, amplification, or ligand-dependent signaling, has been implicated in malignant progression, invasive spread, and therapeutic failure across multiple malignancies, including lung cancer [4,5,6]. Activation of c-MET initiates multiple intracellular signaling pathways, including PI3K/AKT signaling, which plays a pivotal role in promoting cellular survival and preventing apoptotic cell death [7,8]. However, the clinical significance of c-MET overexpression and its functional relationship with AKT activation in lung cancer remains incompletely defined [6].

Previous studies have reported variable frequencies of c-MET dysregulation in lung cancer and inconsistent associations with patient outcomes [9,10]. Therapeutic targeting of the MET pathway has emerged as a promising strategy in tumors harboring MET alterations. Several MET tyrosine kinase inhibitors (TKIs), including capmatinib and tepotinib, have received regulatory approval for the treatment of advanced NSCLC harboring MET exon 14 skipping mutations and have demonstrated clinically meaningful and durable responses in this molecularly defined patient population [11,12]. In addition, other MET-targeted agents, such as crizotinib, savolitinib, and cabozantinib, have shown therapeutic activity in MET-driven malignancies, including NSCLC, papillary renal cell carcinoma, and gastric cancer [13]. Despite these advances, the clinical benefit of MET-targeted therapies remains largely restricted to selected patient subgroups, and durable responses are frequently limited by the emergence of acquired resistance. Multiple mechanisms contribute to therapeutic failure, including tumor heterogeneity, secondary MET kinase-domain alterations, activation of bypass signaling pathways, and incomplete suppression of downstream survival networks. Notably, persistent activation of the PI3K/AKT signaling pathway has been identified as a key mechanism underlying resistance to MET inhibition and continued tumor cell survival [14]. These findings suggest that c-MET inhibitor monotherapy may be insufficient to achieve sustained therapeutic responses and support the development of rational combination strategies targeting both c-MET and its critical downstream effectors. Accordingly, AKT signaling has emerged as an attractive therapeutic target and a potential mediator of resistance in c-MET-driven cancers [14].

In this study, we investigated the clinical and functional significance of c-MET signaling in lung cancer using a tissue microarray cohort, lung cancer cell lines, and in vivo xenograft models. We examined the association between c-MET expression, AKT activation, and patient survival, and assessed the therapeutic efficacy of pharmacological and genetic inhibition of c-MET, alone and in combination with AKT inhibition. Although c-MET and AKT signaling have individually been implicated in NSCLC biology, the clinical relationship between c-MET overexpression and AKT activation, and the therapeutic potential of their combined inhibition remain incompletely characterized. Furthermore, limited studies have integrated patient-derived clinical data with mechanistic and in vivo therapeutic validation. Therefore, we sought to investigate the association between c-MET expression, AKT activation, and clinical outcome, and to determine whether simultaneous targeting of both pathways could provide enhanced antitumor activity in NSCLC. Our findings provide mechanistic and translational evidence that c-MET-driven AKT activation contributes to tumor progression and supports c-MET–AKT co-targeting as a promising therapeutic strategy in lung cancer.

## 2. Materials and Methods

### 2.1. Cell Culture

Human NSCLC cell lines, A549, COLO-699, and H2030, were purchased from the American Type Culture Collection (ATCC, Manassas, VA, USA). Cells were propagated in RPMI-1640 medium supplemented with 10% FBS and 1% penicillin–streptomycin and maintained at 37 °C in a humidified incubator with 5% CO_2_.

### 2.2. Reagents and Antibodies

The c-MET inhibitor PHA665752 was obtained from Tocris Bioscience (Cabot Park, Bristol, UK). The AKT inhibitor MK2206 was obtained from Selleck Chemicals (Houston, TX, USA). Antibodies against phospho Met (3121), c-MET (3148), Phospho AKT (9271), AKT (9272), Phospho Bad (9291) Caspase-8 (9746), Bid (2002), Bcl-xL (2764), Bcl2 (2762), PARP (9542) and GAPDH (2118) were purchased from Cell Signaling Technology (Danvers, MA, USA). Antibodies against Bax (sc-7480), caspase-9 (sc-17784) and caspase-3 (sc-7272) were obtained from Santa Cruz Biotechnology, Inc. (Santa Cruz, CA, USA). XIAP (610763) and Caspase-8 (51-8084) antibodies were sourced from BD Pharmingen (San Diego, CA, USA). Annexin V-FITC reagent was acquired from Thermo Fisher Scientific (Waltham, MA, USA).

### 2.3. Cell Viability Assay

Cell viability was evaluated using the MTT assay [15]. Briefly, NSCLC cells (5 × 10^3^) were seeded into 96-well plates and allowed to adhere overnight. Cells were then treated with varying concentrations of PHA and MK2206, either alone or in combination, for 48 h. Following treatment, cells were exposed to MTT solution (5 mg/mL; 10 µL/well) and incubated for 4 h at 37 °C. The resulting formazan precipitates were dissolved in DMSO after removal of the culture medium, and absorbance was measured at 490 nm using a microplate reader (Molecular Devices, San Jose, CA, USA). Viability values were normalized to untreated controls and reported as percentages.

### 2.4. Clonogenic Assay

NSCLC cells were seeded at low density (500 cells/well) in 6-well plates and cultured for 8–10 days to allow colony formation. Colonies were fixed with 4% formaldehyde, stained with 2% crystal violet in 10% methanol, and quantified by colony counting. Representative images were captured for documentation.

### 2.5. Annexin V Apoptosis Assay

Apoptosis was evaluated by dual staining with Annexin V-FITC and propidium iodide (PI), followed by flow cytometry analysis [16]. Briefly, NSCLC cells were treated with varying concentrations of PHA-665752 and MK2206, either alone or in combination, for 48 h. After treatment, cells were collected, rinsed with ice-cold PBS, and suspended in Annexin V binding buffer. Following staining with Annexin V-FITC and PI according to the supplier’s recommendations, cells were incubated in the dark at room temperature and analyzed by flow cytometry using a FACSCalibur instrument (BD Biosciences, San Jose, CA, USA). Flow cytometry data were processed using BD CellQuest Pro version 5.2.1 software (BD Biosciences, San Jose, CA, USA). Apoptotic cell populations were quantified based on Annexin V-positive staining.

### 2.6. Cell Lysis and Western Blotting

After treatment, NSCLC cells were collected and lysed using a phosphorylation-preserving lysis buffer as previously reported [17]. Protein concentrations were quantified using the Bio-Rad protein assay kit. Equivalent amounts of total protein (10–20 μg) were separated by SDS–polyacrylamide gel electrophoresis and transferred onto PVDF membranes (Immobilon, Millipore, Billerica, MA, USA). Membranes were blocked to prevent nonspecific binding and incubated overnight at 4 °C with the indicated primary antibodies. Following washing steps, HRP-linked anti-rabbit or anti-mouse secondary antibodies (Cell Signaling Technology) were applied. Immunoreactive bands were visualized using an enhanced chemiluminescence (ECL) substrate (Amersham, Piscataway, NJ, USA) and captured using a chemiluminescence imaging system. Full-length, uncropped immunoblot images are provided in Supplementary Figure S1.

### 2.7. siRNA Transfection

Transient silencing of c-MET was achieved via siRNA transfection mediated by Lipofectamine™ 2000 (Invitrogen, Carlsbad, CA, USA) following the supplier’s recommended protocol. Briefly, cells were seeded in 6-well plates and allowed to reach appropriate confluency prior to transfection. Cells were then transfected with c-MET-specific siRNA at concentrations of 50 nM and 100 nM, along with a scrambled siRNA control (100 nM) (Santa Cruz Biotechnology, Santa Cruz, CA, USA). Forty-eight hours after transfection, protein lysates were prepared, and c-MET suppression was verified by immunoblotting.

### 2.8. Animals and Xenografts Study

Female NU/J athymic mice aged six weeks were obtained from Jackson Laboratory (Bar Harbor, ME, USA) and allowed to acclimate to the animal facility for a minimum of seven days before experimentation in a sterile, pathogen-free facility under controlled environmental conditions (12 h light/dark cycle) with ad libitum access to food and water. Animals were maintained under standard environmental enrichment conditions. All animal procedures were conducted in accordance with institutional guidelines and were approved by the Animal Care and Use Committee (ACUC) of King Faisal Specialist Hospital and Research Center (RAC#2120021; Approval Date: 16 September 2018). For xenograft generation, A549 cells (4 × 10^6^ cells per mouse) were suspended in serum-free medium and mixed 1:1 with Matrigel basement membrane matrix. The cell suspension was subcutaneously injected into the flanks of female NU/J mice. Once tumors became established, mice were randomly assigned to four treatment groups (*n* = 4 mice per group; total *n* = 16): (1) vehicle control, (2) PHA, (3) MK2206, and (4) PHA + MK2206 combination treatment. Sample sizes were selected based on previous comparable xenograft studies and institutional ethical considerations. Once tumors reached approximately 100 mm^3^, mice were treated with PHA (20 mg·kg^−1^, i.p.) and MK2206 (120 mg·kg^−1^, oral gavage), administered either as single agents or in combination, three times per week for 30 days. Vehicle controls consisted of 0.1% DMSO (i.p.) for PHA and 30% Captisol (oral) for MK2206. Tumor progression was assessed through serial tumor measurements, while body weights were monitored weekly as an indicator of treatment tolerability. Animals were monitored regularly for signs of distress and euthanized according to institutional humane endpoint guidelines. Blinding was not performed during treatment allocation or tumor assessment. No animals were excluded from the study or omitted from the final analysis. At four weeks post-inoculation, mice were euthanized by CO_2_ inhalation followed by cervical dislocation. Tumors were excised, weighed, and immediately snap-frozen in liquid nitrogen for subsequent molecular analyses.

### 2.9. Patient Sample Selection

A total of 97 formalin-fixed, paraffin-embedded (FFPE) primary lung cancer specimens were obtained from patients diagnosed at King Faisal Specialist Hospital and Research Centre (Riyadh, Saudi Arabia) between 1990 and 2015. Archived tissue blocks were retrospectively selected from the pathology repository for the construction of tissue microarrays. Eligible cases were required to have histopathological confirmation of lung cancer, sufficient tumor material for analysis, and available clinical follow-up information. Ethical approval for the study was granted by the Institutional Review Board and Research Advisory Council (RAC#2120021), and the requirement for informed consent was waived due to the retrospective use of archived specimens.

### 2.10. Tissue Microarray (TMA) Construction and Immunohistochemistry (IHC) Analysis

To construct tissue microarrays, representative tumor areas were identified in 97 FFPE lung cancer specimens. Duplicate 0.6 mm tissue cores were sampled from each donor block and transferred into recipient paraffin blocks using a semiautomated tissue arrayer (Beecher Instruments, Woodland, WI, USA).

TMA sections were processed and stained following established protocols. Slides were incubated with primary antibodies against c-MET (polyclonal, 44888G, 1:500, pH 9.0; Invitrogen) and p-AKT (Ser473, pre-diluted, pH 9.0; Cell Signaling Technology). Immunoreactivity was visualized using the Dako EnVision+ detection system (Agilent Technologies, Glostrup, Denmark) and 3,3′-diaminobenzidine (DAB) as the chromogenic substrate. Following signal development, sections were counterstained with hematoxylin, dehydrated through graded alcohols, and cover slipped. Negative control sections were processed in parallel with omission of the primary antibody, while normal tissue samples served as control tissues. To ensure consistency, freshly cut sections were stained simultaneously. c-MET expression was evaluated using the H-score. The intensity of staining was scored from 0–3 (0—absent, 1+: weak, 2+: moderate, 3+: strong), and the proportion of tumor cells staining at that particular intensity was recorded as 5% increments from a range of 0–100. A final H score was assigned using the following formula: H score = [1 × (% cells 1+) + 2 × (% cells 2+) + 3 × (% cells 3+)]. This H score ranges from 0–300. Two scores per tumor were analyzed in order to minimize the number of missing/un-interpretable spots. However, the higher of the two scores was used as the final score. X-tile plots were constructed for assessment of biomarker and optimization of cutoff points based on outcome as described earlier [18]. Based on X-tile plots, lung cancer cases were classified into two subgroups: those with H score ≤ 100 were defined as low expression of c-MET and those with H score > 100 were defined as high expression. p-AKT expression was evaluated using a previously established scoring system [19]. Staining intensity was graded from 0 to 3, with scores of 0–1 considered p-AKT-negative and scores of 2–3 considered p-AKT-positive for subsequent statistical analyses.

### 2.11. Statistical Analysis

Associations between protein expression and clinicopathological parameters were assessed using chi-square analysis. Overall survival probabilities were estimated by the Kaplan–Meier method and compared using the Mantel–Cox log-rank test. Statistical significance was defined as a two-sided *p* value < 0.05. All analyses were carried out using JMP 14.0 software (SAS Institute, Cary, NC, USA).

For functional studies, data are presented as the mean ± SD from three independent biological experiments (*n* = 3), with the number of technical replicates specified for each assay. Differences among groups were evaluated using one-way ANOVA with Tukey’s post hoc correction in IBM SPSS Statistics version 21 (IBM Corp., Armonk, NY, USA). A two-sided *p* value < 0.05 was considered statistically significant.

## 3. Results

### 3.1. c-MET Immunoexpression and Association with Clinicopathological Characteristics

**Figure 1:**
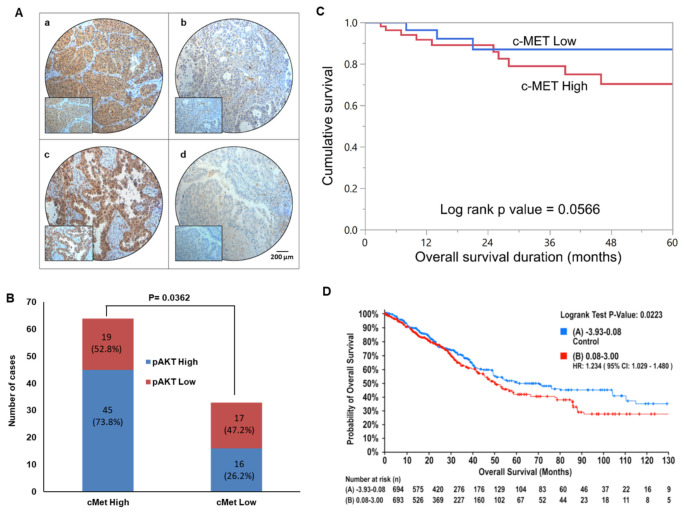
(**A**) Tissue microarray-based immunohistochemical analysis of c-MET and p-AKT expression in lung cancer specimens. Representative images showing high expression of (**a**) c-MET and (**c**) p-AKT, and low expression of (**b**) c-MET and (**d**) p-AKT. Images were captured at 20× magnification (objective 20×/0.70), with inset images at 40× magnification (objective 40×/0.85) using an Olympus BX51 microscope (Olympus America Inc., Center Valley, PA, USA, scale bar = 200 μm). (**B**) Stacked bar graph showing the distribution of p-AKT expression across c-MET-high and -low groups. A higher proportion of p-AKT-positive cases was observed in the c-MET-high group. A significant association between c-MET expression and p-AKT status was identified (Chi-square test, *p* = 0.0362). (**C**,**D**) Kaplan–Meier survival analysis showing a trend toward poorer overall survival in the TMA cohort with high c-MET expression compared to low expression (*p* = 0.0566), and a statistically significant association between high MET expression and reduced overall survival in the TCGA LUAD dataset (*p* = 0.0223). TCGA analysis was performed using MET mRNA expression stratified by cBioPortal z-score thresholds. Survival significance was determined by the log-rank test. HR, hazard ratio; CI, confidence interval.

The expression of c-MET and p-AKT was evaluated in lung cancer tissue microarrays using immunohistochemical staining. c-MET expression was predominantly localized to the cytoplasm of tumor cells. Overall, c-MET overexpression was observed in 66% (64/97) of cases (Figure 1A,B). Statistical analysis revealed a significant association between c-MET overexpression and increased p-AKT expression (*p* = 0.0362; Figure 1B).

Survival analysis of the TMA cohort demonstrated that patients with c-MET-overexpressing tumors exhibited a trend toward poorer overall 5-year survival compared to those with low c-MET expression; however, statistical significance was not achieved (log-rank *p* = 0.0566; Figure 1C).

### 3.2. High MET Expression Correlates with Poor Overall Survival in an Independent Cohort

To further evaluate the prognostic significance of MET expression, we analyzed overall survival in lung adenocarcinoma (LUAD) patients from the TCGA dataset. Patients with high MET expression exhibited significantly poorer overall survival than those with low MET expression, as demonstrated by Kaplan–Meier analysis (log-rank *p* = 0.0223; HR = 1.234, 95% CI: 1.029–1.480; Figure 1D). Patients with high MET expression exhibited consistently lower survival probabilities compared to those with low expression. The number of patients at risk was comparable between groups at baseline, and survival curves progressively diverged over time. These findings support the prognostic relevance of MET expression and indicate that increased MET abundance is linked with adverse clinical outcomes in LUAD.

### 3.3. Down-Regulation of c-MET Inhibited Lung Cancer Cell Growth In Vitro

**Figure 2:**
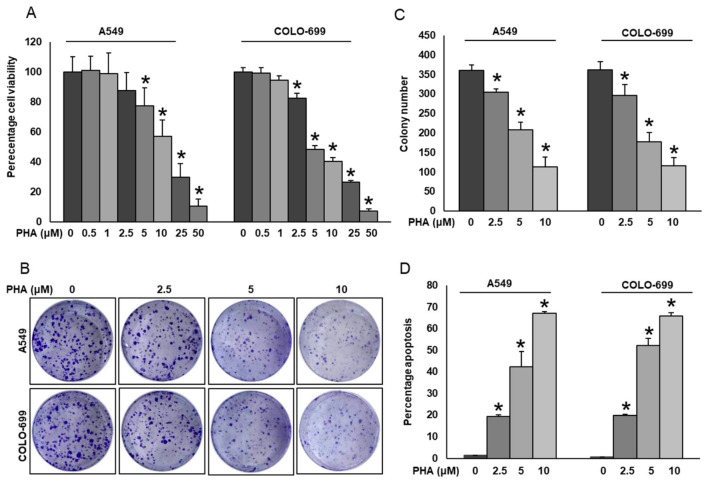
Pharmacological inhibition of c-MET suppresses NSCLC cell growth and promotes apoptosis in vitro. (**A**) Effects of PHA on cell viability. NSCLC cells (5 × 10^3^ cells/well) were exposed to the indicated concentrations of PHA for 48 h, and cell viability was determined using the MTT assay. (**B**,**C**) Effects of PHA on colony-forming ability. Following treatment, NSCLC cells (5 × 10^2^ cells) were plated in 6-well culture dishes and maintained for an additional 10 days. Colonies were then fixed, stained with crystal violet, and quantified. (**D**) Induction of apoptosis by PHA. Cells were treated with the indicated concentrations of PHA for 48 h, stained with Annexin V-FITC, and analyzed by flow cytometry to determine apoptotic cell populations. Quantitative results are shown as mean ± SD derived from three independent experiments. * *p* < 0.05 versus the untreated control group.

Based on the clinical association between c-MET expression and patient outcome, we next investigated the biological consequences of c-MET inhibition in NSCLC cells. Our clinical data showed the overexpression of c-MET in lung cancer cases by immunohistochemistry. We therefore examined whether selective pharmacological blockade of c-MET could suppress NSCLC cell growth under in vitro conditions. NSCLC cell lines (A549 and COLO-699) were incubated with and without indicated doses of PHA, a c-MET selective inhibitor, for 48 h; cell viability was determined by MTT assay. As shown in Figure 2A, PHA caused a significant (*p* < 0.05) dose-dependent growth inhibition in both cell lines. A similar result was observed in a clonogenic assay (Figure 2B), where the treatment of PHA (0, 2.5, 5 and 10 μM) significantly (*p* < 0.05) decreased colony formation in a dose-dependent manner (Figure 2B,C). To determine whether the observed growth inhibition was associated with apoptosis, A549 and COLO-699 cells were treated with PHA (2.5, 5, and 10 μM) for 48 h and analyzed by Annexin V/propidium iodide staining followed by flow cytometry. As shown in Figure 2D, PHA treatment significantly increased the proportion of apoptotic cells in both cell lines, indicating that the reduction in cell viability is primarily due to induction of apoptosis.

### 3.4. c-MET Down-Regulation Leads to Inactivation of AKT and Induces Caspase-Dependent Mitochondrial Apoptotic Pathway

**Figure 3:**
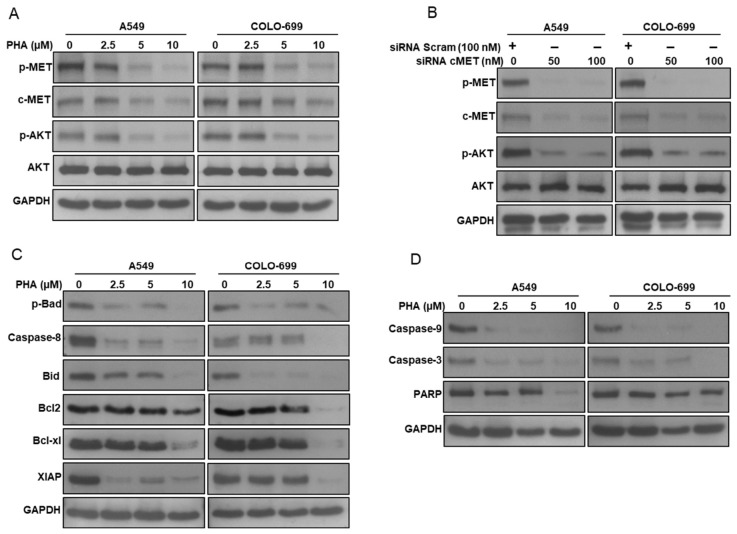
c-MET inhibition suppresses AKT signaling and activates apoptotic pathways in NSCLC cells. (**A**) Effects of PHA on c-MET and AKT signaling. A549 and COLO-699 cells were exposed to increasing concentrations of PHA (2.5, 5, and 10 μM) for 48 h. Whole-cell lysates were prepared, and protein expression levels of p-MET, total MET, p-AKT, total AKT, and GAPDH were examined by immunoblot analysis. (**B**) Effects of c-MET silencing on downstream signaling. A549 and COLO-699 cells were transfected with scrambled control siRNA (100 nM) or c-MET-targeting siRNA (50 and 100 nM) using Lipofectamine 2000. Protein lysates were analyzed by Western blotting for p-MET, total MET, p-AKT, total AKT, and GAPDH. (**C**) Modulation of apoptosis-related proteins following c-MET inhibition. A549 and COLO-699 cells were treated with PHA (2.5–10 μM) for 48 h. Cell lysates were subjected to immunoblotting to assess the expression of phospho-Bad, caspase-8, Bid, Bcl-2, Bcl-xL, XIAP, and GAPDH. (**D**) Activation of the caspase cascade by PHA. NSCLC cells were treated with the indicated concentrations of PHA for 48 h. Protein extracts were analyzed for caspase-9, caspase-3, PARP, and GAPDH expression by Western blotting.

Given the observed association between c-MET overexpression and AKT activation in clinical samples, we next examined whether c-MET inhibition modulates AKT signaling and downstream apoptotic pathways. Treatment with PHA resulted in marked inactivation of both c-MET and AKT in A549 and COLO-699 cells, which was further confirmed by c-MET siRNA-mediated knockdown (Figure 3A,B). Inhibition of AKT signaling was accompanied by reduced phosphorylation of Bad, indicating activation of apoptotic signaling (Figure 3C). To further delineate the mechanism, we evaluated key components of the mitochondrial apoptotic pathway. PHA induced activation of caspase-8 and cleavage of Bid, along with downregulation of anti-apoptotic proteins Bcl-2, Bcl-xL, and XIAP (Figure 3C). This was accompanied by activation of downstream apoptotic markers, including cleavage of caspase-9, caspase-3, and PARP (Figure 3D). To further validate the role of caspases in PHA-induced apoptosis, NSCLC cells were pretreated with the pan-caspase inhibitor zVAD-fmk (80 µM) for 3 h prior to treatment with PHA (10 µM) for 48 h. As shown in Figure S2, zVAD-fmk pretreatment markedly attenuated PHA-induced apoptosis compared with cells treated with PHA alone, confirming that apoptosis induced by c-MET inhibition is largely caspase-dependent.

**Figure 4:**
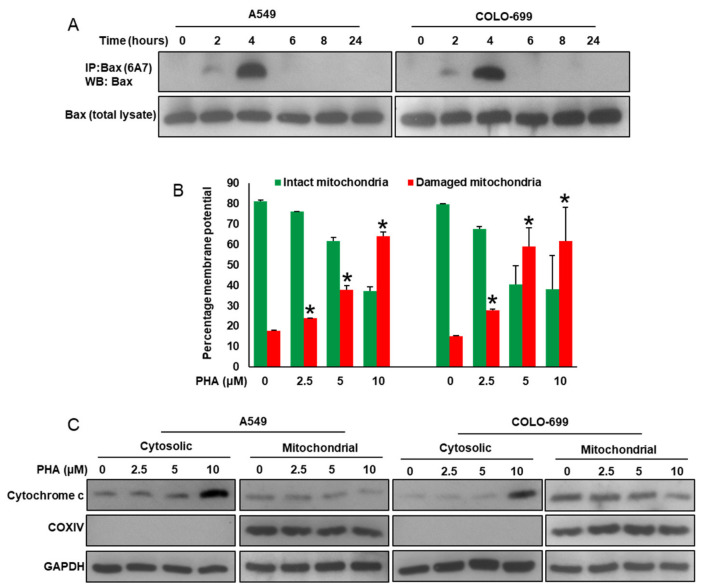
c-MET inhibition promotes mitochondrial apoptotic events in NSCLC cells. (**A**) PHA induces Bax conformational activation. NSCLC cells were exposed to PHA for the indicated time intervals. Following treatment, cell lysates were prepared in 1% CHAPS buffer and subjected to immunoprecipitation using the Bax 6A7 monoclonal antibody, which recognizes the activated conformation of Bax. Immunoprecipitates were analyzed by immunoblotting with a Bax-specific polyclonal antibody. Total Bax expression was determined by Western blot analysis of whole-cell lysates. (**B**) PHA disrupts mitochondrial membrane integrity. NSCLC cells were incubated with the indicated concentrations of PHA for 48 h. Changes in mitochondrial membrane potential were assessed by JC-1 staining followed by flow cytometric analysis. Cells retaining intact mitochondrial membrane potential are shown in green, whereas cells exhibiting membrane depolarization are shown in red. Data are presented as mean ± SD from three independent experiments. * *p* < 0.05 versus untreated controls. (**C**) PHA promotes cytochrome c release from mitochondria. NSCLC cells were treated with PHA for 48 h, after which cytosolic and mitochondrial fractions were isolated. Protein samples were analyzed by Western blotting for cytochrome c expression, with GAPDH used as a loading control.

Consistent with these findings, PHA also triggered Bax conformational activation and disruption of mitochondrial membrane potential, as indicated by JC-1 staining (Figure 4A,B). This was further supported by the release of cytochrome c from mitochondria into the cytosol (Figure 4C), confirming activation of the mitochondrial apoptotic pathway.

### 3.5. Co-Inhibition of c-MET by PHA and AKT by MK2206 Synergistically Inhibited Lung Cancer Cells Growth In Vitro

**Figure 5:**
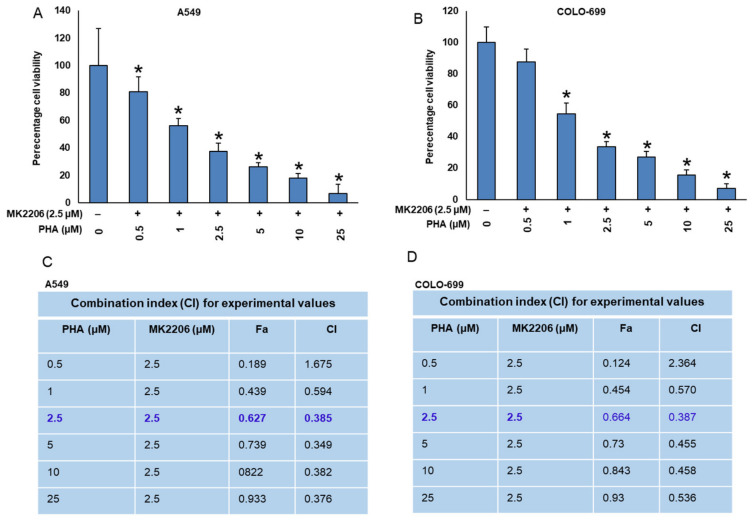
Combined inhibition of c-MET and AKT reduces cell viability and shows synergistic effects in NSCLC cells. (**A**,**B**) Cell viability of A549 (**A**) and COLO-699 (**B**) cells following treatment with MK2206 (2.5 μM) alone or in combination with increasing concentrations of PHA (0–25 μM) for 48 h, as assessed by MTT assay. Data are presented as percentage cell viability relative to untreated control cells. Error bars represent mean ± SD. (**C**,**D**) Combination index (CI) analysis for A549 (**C**) and COLO-699 (**D**) cells treated with PHA and MK2206 (2.5 μM). Fraction affected (Fa) and CI values were calculated using the Chou–Talalay method. CI < 1 indicates synergism, CI = 1 indicates additive effect, and CI > 1 indicates antagonism. Notably, the combination at 2.5 μM PHA and 2.5 μM MK2206 demonstrated strong synergistic effects in both cell lines. * *p* < 0.05.

Based on the observed association between c-MET and AKT signaling, we hypothesized that dual targeting of these pathways would enhance therapeutic efficacy in NSCLC cells. To evaluate the combined effect of c-MET and AKT inhibition, NSCLC cell lines were treated with varying concentrations of PHA in combination with a suboptimal dose of MK2206. As shown in Figure 5A,B, the combination treatment resulted in a significant reduction in cell viability. Synergistic interactions were assessed using the Chou–Talalay method with CalcuSyn software version 2.11 (Biosoft, Cambridge, UK) [20]. The combination of PHA (2.5 μM) and MK2206 (2.5 μM) demonstrated strong synergism in both cell lines, with combination index (CI) values of 0.385 in A549 cells (Figure 5C and Figure S3A,B) and 0.387 in COLO-699 cells (Figure 5D and Figure S3C,D). To further validate these findings in an independent NSCLC model, we evaluated the combination treatment in H2030 cells. Consistent with our observations in A549 and COLO-699 cells, combined inhibition of c-MET and AKT resulted in significantly greater growth inhibition than either agent alone and demonstrated strong synergism, with a CI value of 0.347 at 2.5 μM PHA and 2.5 μM MK2206, further supporting the therapeutic potential of dual targeting of these pathways in NSCLC (Figure S4A–D).

**Figure 6:**
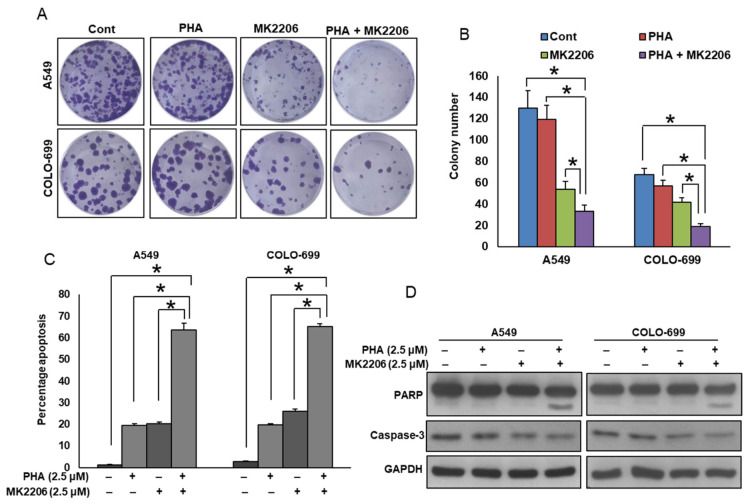
Combined inhibition of c-MET and AKT synergistically suppresses NSCLC cell growth and enhances apoptosis in vitro. (**A**,**B**) Combined treatment with PHA and MK2206 reduces colony-forming capacity. Following exposure to PHA and MK2206, either alone or in combination, NSCLC cells (5 × 10^2^ cells) were plated in 6-well plates and cultured for an additional 10 days to allow colony formation. Colonies were subsequently stained with crystal violet and quantified. Data are presented as mean ± SD from three independent experiments (*n* = 3). * *p* < 0.05. (**C**) Combined c-MET and AKT inhibition promotes apoptotic cell death. NSCLC cells were treated with the indicated concentrations of PHA and MK2206 as single agents or in combination for 48 h. Apoptosis was assessed by Annexin V-FITC/propidium iodide (PI) staining followed by flow cytometric analysis. Quantitative results are shown as mean ± SD derived from three independent experiments. * *p* < 0.05 versus the untreated control group. (**D**) Dual targeting of c-MET and AKT potentiates apoptotic signaling in NSCLC cells. Cells were treated with the indicated doses of PHA and MK2206 for 48 h. Protein lysates were subjected to immunoblot analysis for caspase-3 and PARP, with GAPDH serving as the loading control.

Consistent with these findings, clonogenic assays showed that the combination treatment significantly reduced colony formation compared to either agent alone (Figure 6A,B). To determine whether this enhanced growth inhibition was associated with apoptosis, cells were treated with suboptimal doses of PHA (2.5 μM) and MK2206 (2.5 μM), either alone or in combination, followed by Annexin V/propidium iodide staining and flow cytometric analysis. As shown in Figure 6C, the combination treatment significantly increased apoptotic cell populations compared to single treatments. Furthermore, co-treatment with PHA and MK2206 led to increased cleavage of PARP and caspase-3, confirming activation of apoptotic pathways (Figure 6D). Collectively, these results demonstrate that dual inhibition of c-MET and AKT synergistically suppresses cell growth and enhances apoptosis in NSCLC cells.

### 3.6. Combined Inhibition of c-MET and AKT Delays Tumor Growth In Vivo

**Figure 7:**
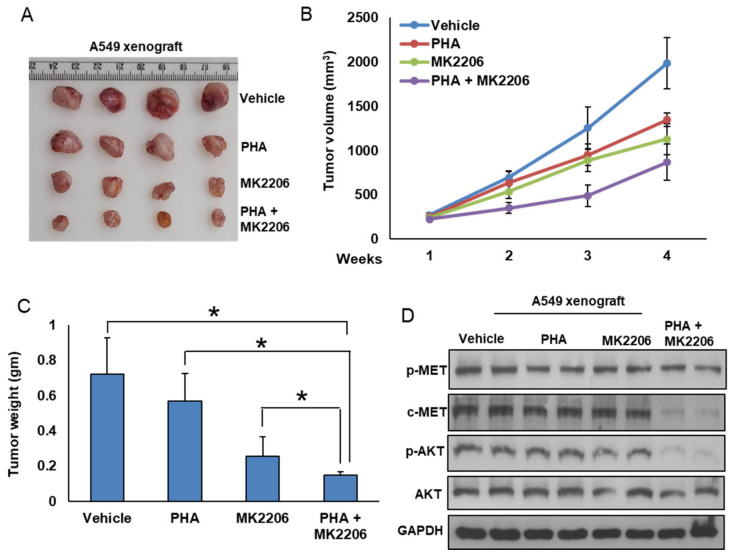
Combined inhibition of c-MET and AKT synergistically inhibits NSCLC tumor growth in vivo. A549 cells (4 × 10^6^ per mouse) were subcutaneously implanted into the flanks of 6-week-old female NU/J mice. Once tumors reached approximately 100 mm^3^, mice were treated with PHA (20 mg·kg^−1^, i.p.) and MK2206 (120 mg·kg^−1^, oral gavage), administered either as single agents or in combination, three times per week for 30 days. Vehicle controls consisted of 0.1% DMSO (i.p.) for PHA and 30% Captisol (oral) for MK2206. (**A**) Representative photographs of excised tumors collected from each treatment group. (**B**) Tumor growth was monitored throughout the study by weekly measurement of tumor volume. Data are presented as the mean tumor volume ± SD for each treatment group (*n* = 4 mice per group). (**C**) At the end of the 4-week treatment period, tumors were harvested and weighed. Mean tumor weights are shown as ± SD for each experimental group. * *p* < 0.05 versus the control group. (**D**) Tumor tissue lysates were analyzed by Western blotting to determine the expression levels of p-MET, total MET, p-AKT, total AKT, and GAPDH.

Building on our in vitro findings demonstrating synergistic suppression of NSCLC cell growth through dual inhibition of c-MET and AKT, we next evaluated the therapeutic efficacy of this combination in vivo using an A549 xenograft model. A549 cells (4 × 10^6^ per mouse) were subcutaneously implanted into the flanks of 6-week-old female NU/J mice. Once tumors reached approximately 100 mm^3^, mice were treated with PHA (20 mg·kg^−1^, i.p.) and MK2206 (120 mg·kg^−1^, oral gavage), administered either as single agents or in combination, three times per week for 30 days. Vehicle controls consisted of 0.1% DMSO (i.p.) for PHA and 30% Captisol (oral) for MK2206. Tumor growth was evaluated in four treatment groups (*n* = 4 mice per group, total *n* = 16). No animals were excluded from the analysis. Combination treatment resulted in a significant delay in tumor growth compared with monotherapy groups, as evidenced by reduced tumor volume and size (Figure 7A,B) and decreased tumor weight at endpoint (Figure 7C). Notably, no significant changes in body weight were observed, indicating acceptable tolerability. At the molecular level, co-treatment markedly reduced the expression of p-MET, c-MET, and p-AKT in tumor tissues (Figure 7D), confirming effective pathway inhibition. Collectively, these findings demonstrate that combined targeting of c-MET and AKT enhances antitumor efficacy in vivo and supports the potential of this strategy as a therapeutic approach for NSCLC.

## 4. Discussion

In this study, we demonstrate that c-MET is frequently overexpressed in lung cancer and that its overexpression is significantly associated with AKT pathway activation and adverse clinical outcomes [6,21,22]. While previous studies have independently implicated c-MET and AKT signaling in lung cancer progression, our study integrates clinical, mechanistic, and therapeutic evidence to establish a link between c-MET overexpression, AKT activation, and therapeutic susceptibility in NSCLC. Using a combination of tissue-based analysis, in vitro functional studies, and in vivo validation, we demonstrate that c-MET inhibition suppresses AKT signaling and that concurrent targeting of both pathways produces synergistic antitumor effects, supporting c-MET–AKT co-targeting as a rational therapeutic strategy in NSCLC.

Our tissue microarray analysis revealed c-MET overexpression in approximately two-thirds of lung cancer cases, consistent with previous reports highlighting the prevalence of MET dysregulation in this disease [9,10]. Importantly, c-MET overexpression was significantly associated with increased p-AKT expression, supporting a functional link between c-MET signaling and activation of the PI3K/AKT pathway in clinical tumor samples [5,7,8]. In our study, c-MET overexpression was associated with a trend toward poorer overall survival in the TMA cohort, although this did not reach statistical significance. However, analysis of the independent TCGA LUAD dataset revealed a statistically significant association between high MET expression and reduced survival. These findings support the prognostic relevance of c-MET in lung cancer, consistent with previous reports [1,6,21]. The lack of statistical significance in the TMA cohort may be attributed to the relatively smaller sample size and cohort heterogeneity, whereas the larger TCGA dataset provided sufficient power to detect a significant association.

Functional studies in lung cancer cell lines further established the oncogenic role of c-MET signaling. Pharmacological inhibition of c-MET using PHA significantly reduced cell proliferation and induced caspase-dependent mitochondrial apoptosis, indicating that lung cancer cells rely on c-MET signaling for survival [5,23]. Consistently, both pharmacological inhibition and siRNA-mediated knockdown of c-MET resulted in marked inactivation of AKT, providing mechanistic evidence that AKT is a critical downstream effector of c-MET-driven oncogenic signaling in lung cancer [5,7].

Notably, combined inhibition of c-MET and AKT using PHA and MK2206 resulted in synergistic suppression of cell growth and enhanced induction of apoptosis compared with either agent alone [24]. This synergy was also observed in vivo, where co-inhibition significantly delayed xenograft tumor growth [25]. These findings suggest that AKT signaling contributes to adaptive survival mechanisms following c-MET inhibition and that dual targeting of this axis can overcome compensatory signaling to achieve improved antitumor efficacy [13]. Importantly, the observed synergistic activity was reproducible across multiple NSCLC models and was further validated in an independent H2030 cell line, supporting the robustness and potential translational relevance of this combination approach. These findings extend previous reports of MET-directed therapy by providing evidence that concurrent inhibition of a key downstream survival pathway can enhance therapeutic efficacy beyond that achieved with c-MET inhibition alone.

The limited clinical success of c-MET inhibitors as monotherapies in lung cancer has highlighted the need for rational combination strategies [23,26,27]. Our data provide strong preclinical support for combined c-MET and AKT inhibition, offering a mechanistic rationale for targeting both upstream receptor signaling and downstream survival pathways [6]. This approach may be particularly relevant in patients with c-MET-overexpressing tumors exhibiting active AKT signaling, a subgroup that may be at higher risk of poor clinical outcomes [6,22].

Several limitations of this study should be acknowledged. While our findings establish a strong association between c-MET overexpression and AKT activation, further validation in independent patient cohorts and integration with genomic alterations such as MET amplification or mutation would strengthen clinical translation [6,9]. The relatively small TMA cohort may have limited the statistical power for survival analysis. In addition, although both pharmacological and genetic inhibition of c-MET consistently suppressed AKT phosphorylation and induced apoptosis, the causal role of AKT in mediating c-MET inhibitor-induced apoptosis was not directly confirmed using constitutively active AKT or AKT overexpression rescue experiments. Such studies would provide additional mechanistic validation of the c-MET–AKT signaling axis and represent an important direction for future investigation. Furthermore, although synergistic activity was confirmed in multiple NSCLC models, broader dose–response matrices, evaluation of additional drug-ratio combinations, and validation across a larger panel of genetically diverse NSCLC models would provide additional translational insight. Future studies should also explore the interaction between c-MET signaling and other resistance pathways, including EGFR- and immune-related mechanisms, to further refine patient selection strategies and identify additional combination approaches [13,28].

## 5. Conclusions

Our study identifies c-MET overexpression as a frequent event in lung cancer that is associated with AKT activation and poor patient survival. We demonstrate that targeting c-MET, particularly in combination with AKT inhibition, effectively suppresses tumor growth and induces apoptosis in preclinical models. These findings support c-MET–AKT co-targeting as a promising therapeutic strategy and provide a strong rationale for future clinical trials aimed at improving outcomes for patients with lung cancer.

## Data Availability

The original contributions presented in this study are included in the article/Supplementary Materials. Further inquiries can be directed to the corresponding author.

## Supplementary Materials

The following supporting information can be downloaded at: https://www.mdpi.com/article/10.3390/cells15131155/s1, Figure S1: Uncropped Western blot images; Figure S2: Effect of universal caspase inhibitor z-VAD/fmk on PHA-induced apoptosis in NSCL cells; Figure S3: Synergistic interaction between c-MET and AKT inhibitors in A549 and Colo-699 cells. Figure S4: Combined inhibition of c-MET and AKT demonstrates synergistic antiproliferative effects in H2030 NSCLC cells.

## References

[1] Bray F., Laversanne M., Sung H., Ferlay J., Siegel R.L., Soerjomataram I., Jemal A. (2024). Global cancer statistics 2022: GLOBOCAN estimates of incidence and mortality worldwide for 36 cancers in 185 countries. CA Cancer J. Clin..

[2] Thai A., Solomon B., Sequist L.V., Gainor L., Heist J.F. (2021). Lung cancer. Lancet.

[3] Sung H., Ferlay J., Siegel R.L., Laversanne M., Soerjomataram I., Jemal A., Bray F. (2021). Global cancer statistics 2020: GLOBOCAN estimates of incidence and mortality worldwide for 36 cancers in 185 countries. CA Cancer J. Clin..

[4] Zhang Y., Xia M., Jin K., Wang S., Wei H., Fan C., Wu Y., Li X., Li X., Li G. (2018). Function of the c-Met receptor tyrosine kinase in carcinogenesis and associated therapeutic opportunities. Mol. Cancer.

[5] Guo R., Luo J., Chang J., Rekhtman N., Arcila M., Drilon A. (2020). MET-dependent solid tumours—Molecular diagnosis and targeted therapy. Nat. Rev. Clin. Oncol..

[6] Drilon A., Cappuzzo F., Ou S.-H.I., Camidge D.R. (2017). Targeting MET in lung cancer: Will expectations finally be MET?. J. Thorac. Oncol..

[7] Organ S.L., Tsao M.-S. (2011). An overview of the c-MET signaling pathway. Ther. Adv. Med. Oncol..

[8] Trusolino L., Bertotti A., Comoglio P.M. (2010). MET signalling: Principles and functions in development, organ regeneration and cancer. Nat. Rev. Mol. Cell Biol..

[9] Zhan S., Li J., Cheng B., Li C., Feng Y., Fan L., Xiong S., Zeng W., Cai Q., Xiang Y. (2024). Landscape of C-MET overexpression in non-small cell lung cancer: A large-scale study of clinicomolecular features and prognosis based on Chinese data. Ther. Adv. Med. Oncol..

[10] Bubendorf L., Dafni U., Schöbel M., Finn S.P., Tischler V., Sejda A., Marchetti A., Thunnissen E., Verbeken E.K., Warth A. (2017). Prevalence and clinical association of MET gene overexpression and amplification in patients with NSCLC: Results from the European Thoracic Oncology Platform (ETOP) Lungscape project. Lung Cancer.

[11] Wolf J., Seto T., Han J.-Y., Reguart N., Garon E.B., Groen H.J., Tan D.S., Hida T., de Jonge M., Orlov S.V. (2020). Capmatinib in MET exon 14–mutated or MET-amplified non–small-cell lung cancer. N. Engl. J. Med..

[12] Paik P.K., Felip E., Veillon R., Sakai H., Cortot A.B., Garassino M.C., Mazieres J., Viteri S., Senellart H., Van Meerbeeck J. (2020). Tepotinib in non–small-cell lung cancer with MET exon 14 skipping mutations. N. Engl. J. Med..

[13] Mo H.-N., Liu P. (2017). Targeting MET in cancer therapy. Chronic Dis. Transl. Med..

[14] Recondo G., Bahcall M., Spurr L.F., Che J., Ricciuti B., Leonardi G.C., Lo Y.-C., Li Y.Y., Lamberti G., Nguyen T. (2020). Molecular mechanisms of acquired resistance to MET tyrosine kinase inhibitors in patients with MET exon 14–mutant NSCLC. Clin. Cancer Res..

[15] Pratheeshkumar P., Raphael T.J., Kuttan G. (2012). Nomilin inhibits metastasis via induction of apoptosis and regulates the activation of transcription factors and the cytokine profile in B16F-10 cells. Integr. Cancer Ther..

[16] Turcios L., Vilchez V., Acosta L.F., Poyil P., Butterfield D.A., Mitov M., Marti F., Gedaly R. (2017). Sorafenib and FH535 in combination act synergistically on hepatocellular carcinoma by targeting cell bioenergetics and mitochondrial function. Dig. Liver Dis..

[17] Siraj A.K., Pratheeshkumar P., Parvathareddy S.K., Divya S.P., Al-Dayel F., Tulbah A., Ajarim D., Al-Kuraya K.S. (2018). Overexpression of PARP is an independent prognostic marker for poor survival in Middle Eastern breast cancer and its inhibition can be enhanced with embelin co-treatment. Oncotarget.

[18] Camp R.L., Dolled-Filhart M., Rimm D.L. (2004). X-tile: A new bio-informatics tool for biomarker assessment and outcome-based cut-point optimization. Clin. Cancer Res..

[19] Uddin S., Ahmed M., Hussain A., Assad L., Al-Dayel F., Bavi P., Al-Kuraya K.S., Munkarah A. (2010). Cyclooxygenase-2 inhibition inhibits PI3K/AKT kinase activity in epithelial ovarian cancer. Int. J. Cancer.

[20] Chou T.-C., Talalay P. (1984). Quantitative analysis of dose-effect relationships: The combined effects of multiple drugs or enzyme inhibitors. Adv. Enzym. Regul..

[21] Kim J.H., Kim H.S., Kim B.J. (2018). Prognostic value of MET copy number gain in non-small-cell lung cancer: An updated meta-analysis. J. Cancer.

[22] Yu H.A., Arcila M.E., Rekhtman N., Sima C.S., Zakowski M.F., Pao W., Kris M.G., Miller V.A., Ladanyi M., Riely G.J. (2013). Analysis of tumor specimens at the time of acquired resistance to EGFR-TKI therapy in 155 patients with EGFR-mutant lung cancers. Clin. Cancer Res..

[23] Reungwetwattana T., Liang Y., Zhu V., Ou S.-H.I. (2017). The race to target MET exon 14 skipping alterations in non-small cell lung cancer: The why, the how, the who, the unknown, and the inevitable. Lung Cancer.

[24] Cheng H., Shcherba M., Pendurti G., Liang Y., Piperdi B., Perez-Soler R. (2014). Targeting the PI3K/AKT/mTOR pathway: Potential for lung cancer treatment. Lung Cancer Manag..

[25] Puri N., Salgia R. (2008). Synergism of EGFR and c-Met pathways, cross-talk and inhibition, in non-small cell lung cancer. J. Carcinog..

[26] Salgia R. (2017). MET in lung cancer: Biomarker selection based on scientific rationale. Mol. Cancer Ther..

[27] Han Y., Yu Y., Miao D., Zhou M., Zhao J., Shao Z., Jin R., Le X., Li W., Xia Y. (2024). Targeting MET in NSCLC: An ever-expanding territory. JTO Clin. Res. Rep..

[28] Nan X., Xie C., Yu X., Liu J. (2017). EGFR TKI as first-line treatment for patients with advanced EGFR mutation-positive non-small-cell lung cancer. Oncotarget.

