# Supplementary material for: c-MET Overexpression Drives AKT Activation, and Combined Inhibition Synergistically Enhances Therapeutic Sensitivity in Non-Small-Cell Lung Cancer
Source: Cells. 2026 Jun 25;15(13):1155. doi: 10.3390/cells15131155 (PMC13360048; doi:10.3390/cells15131155)
Supplement: Supplementary file 1 [file cells-15-01155-s001.zip › cells-4352006-supplementary.pdf]

Figure S1. Uncropped Western blot images

Figure 3A

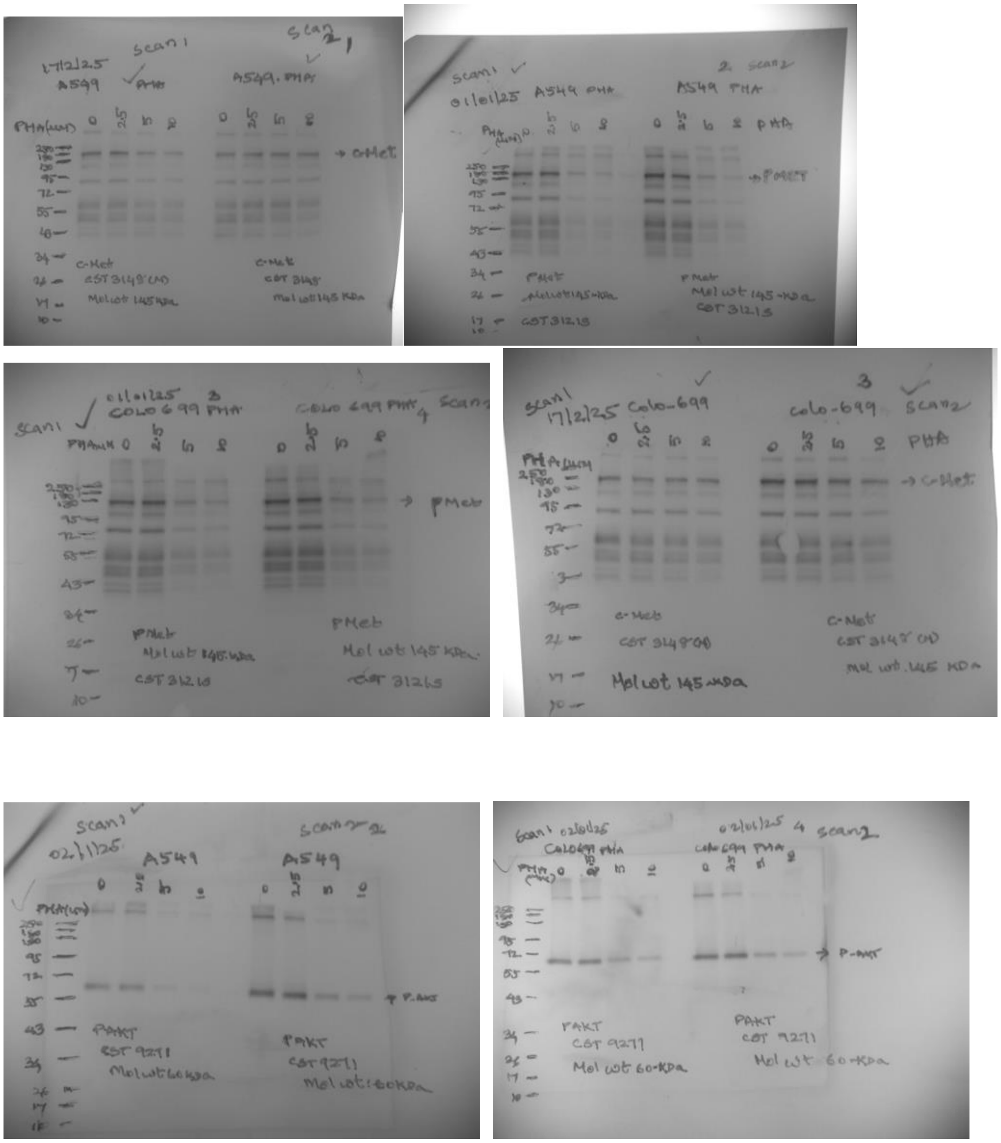

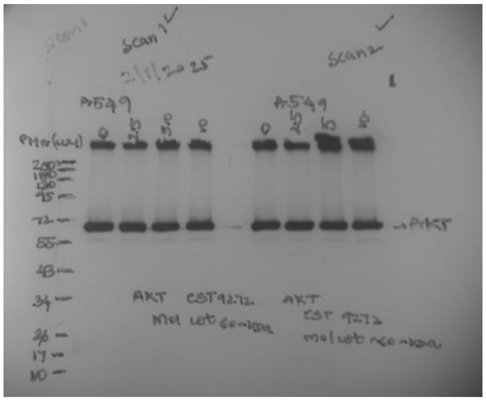
2/1/2025
A549
A549
AKT
CST 9272
AKT
CST 9272

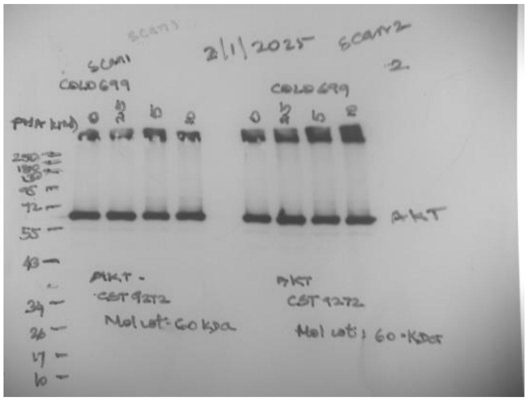
2/1/2025
COLO 699
COLO 699
AKT
CST 9272
AKT
CST 9272

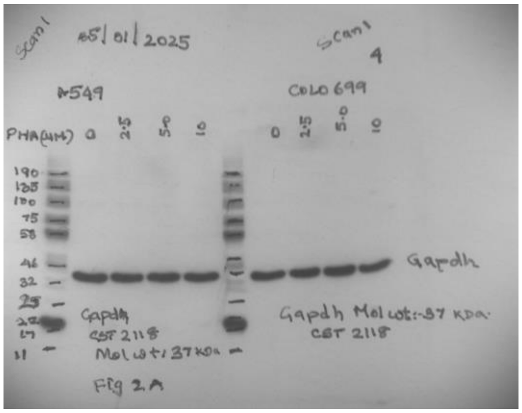
05/01/2025
A549
COLO 699
Gapdh
Gapdh
CST 2118
CST 2118
Fig 2A

Figure 3B

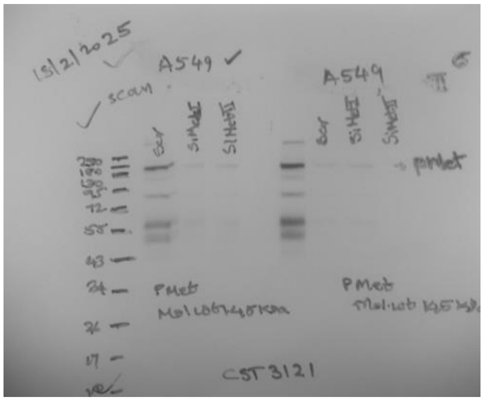
13/2/2025
A549
A549
CST 3121

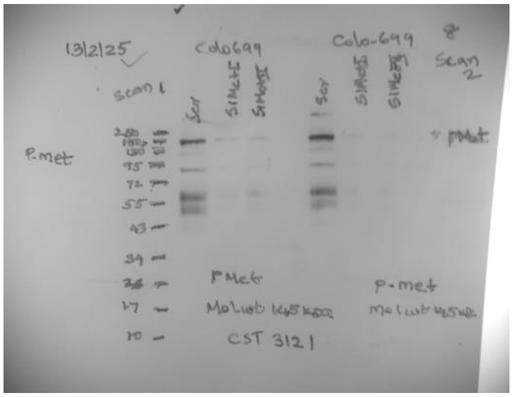
13/2/25
Colo699
Colo-699
CST 3121

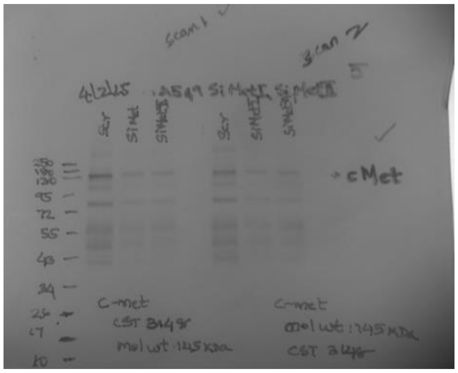
scan 1
scan 2
4/2/25 A549 si Met I, si Met II
Scr
cMet
c-met
CST 3148
mol wt 145 kDa
c-met
mol wt 145 KDa
CST 3148

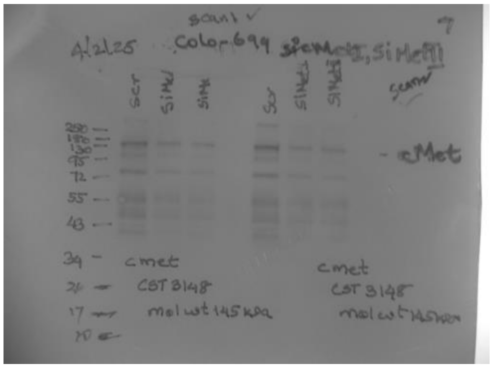
scan 1
4/2/25 Colo 699
Scr
cMet
cmet
CST 3148
mol wt 145 kDa
cmet
CST 3148

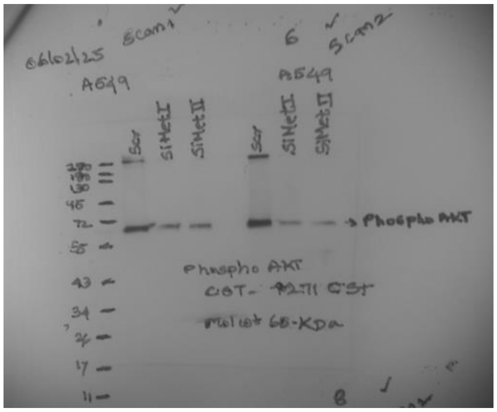
scan 1
scan 2
A549
Scr
Si Met I
Si Met II
A549
Phospho AKT
Phospho AKT
Mol wt 60-KDa

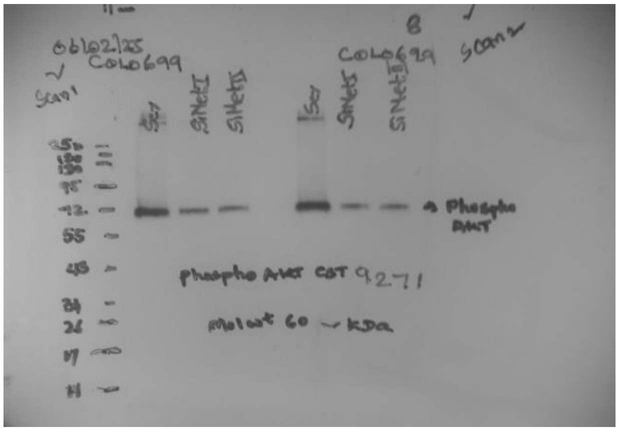
scan 1
COLO699
COLO699
scan 2
Scr
Phospho AKT
Phospho AKT CST 9271
Mol wt 60 KDa

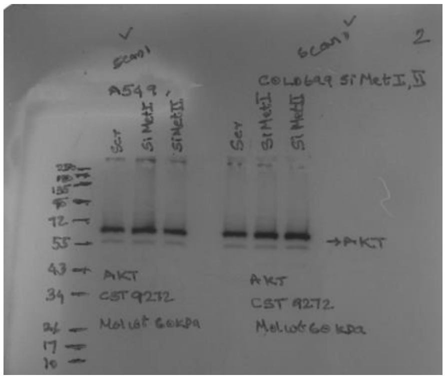
scan 1
scan 1
A549
COLO699 si Met I, II
Scr
Si Met I
Si Met II
AKT
AKT
CST 9272
AKT
CST 9272
Mol wt 60 kDa

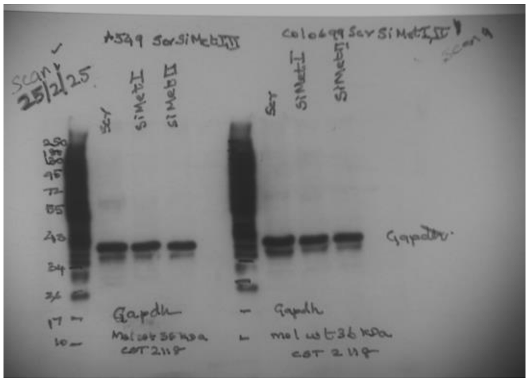
A549 Scr si Met I, II
Colo699 Scr Si Met I, II
scan
25/2/25
Scr
si Met I
si Met II
Gapdh
Gapdh
CST 2118
Gapdh
CST 2118

Figure 3C

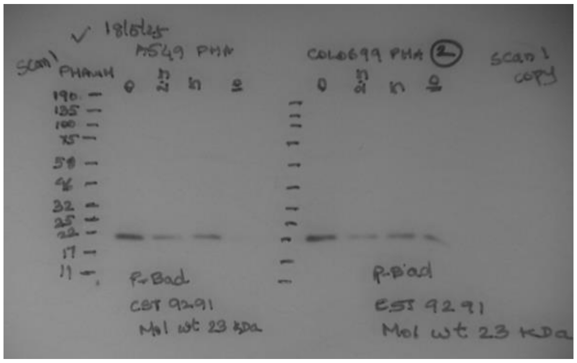
A549 PHA
COLO699 PHA
P-Bad
CST 9291
Mol wt 23 kDa

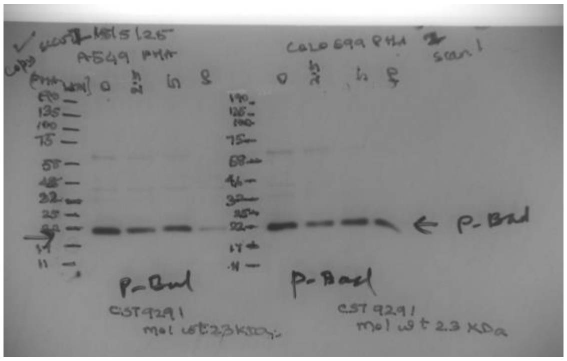
A549 PHA
COLO699 PHA
P-Bad
CST 9291
Mol wt 23 kDa

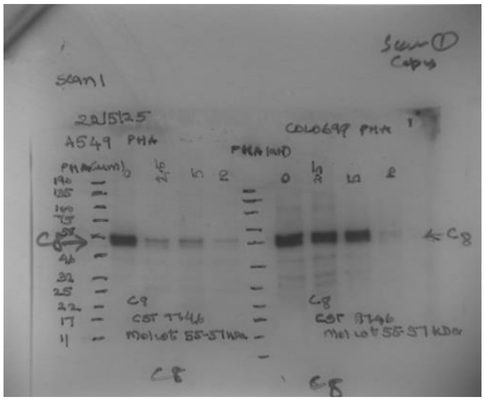
A549 PHA
COLO699 PHA
C8

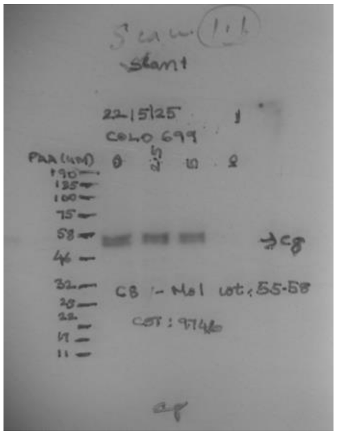
COLO 699
C8

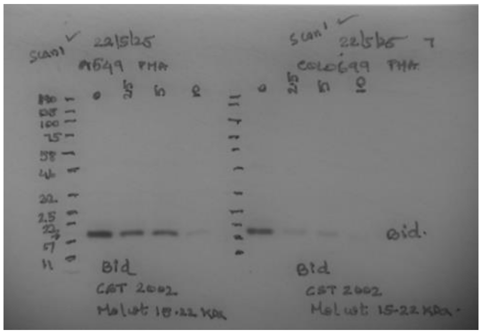
A549 PHA
COLO699 PHA
Bid
CST 2002

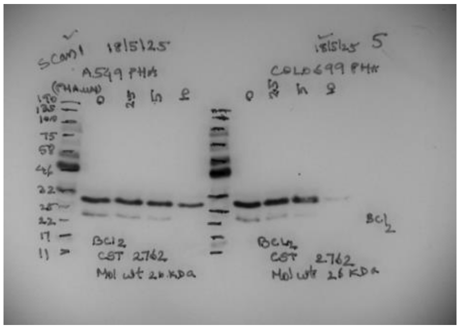
A549 PHA
COLO699 PHA
Bcl2
CST 2762
Mol wt 26 kDa

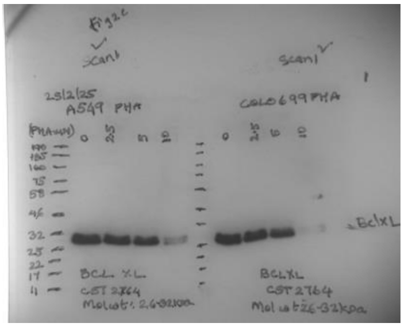
A549 PHA
COLO699 PHA
BCL XL
CST 2764

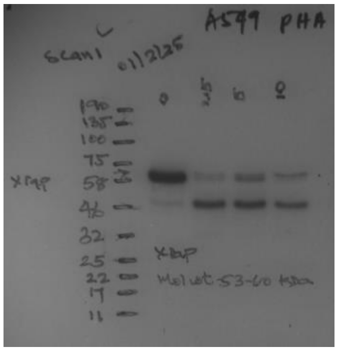
A549 PHA

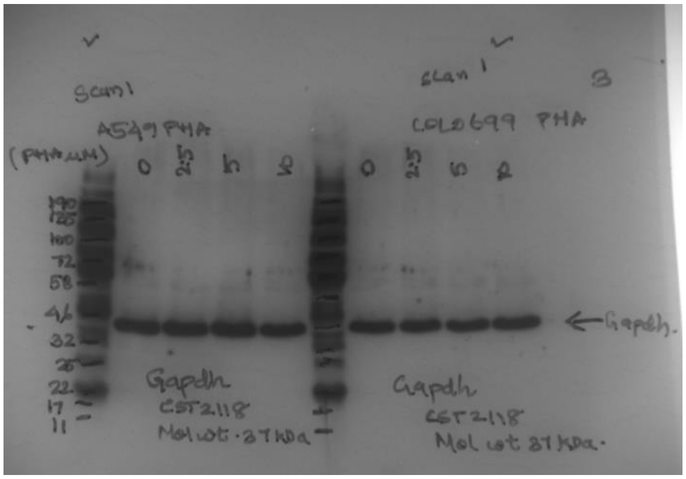
Scan 1
A549 PHA
COLO699 PHA
Gapdh
CST 2118
Mol wt. 37 kDa
Gapdh
CST 2118
Mol wt 37 kDa

Figure 3D

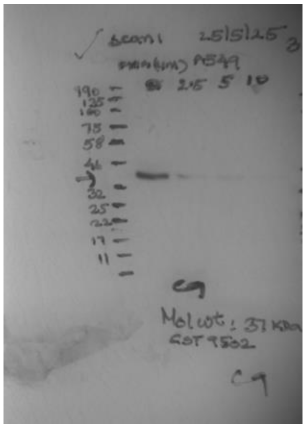
A549
C9
CST 9502

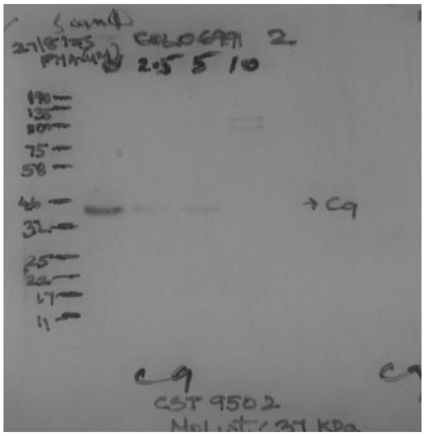
COLO699
C9
CST 9502

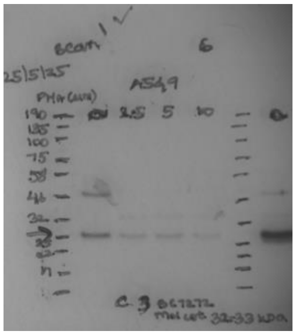
A549

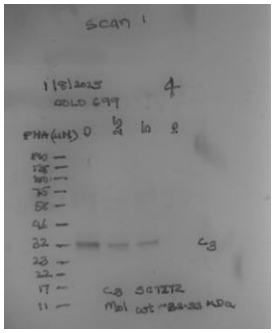
COLO 699

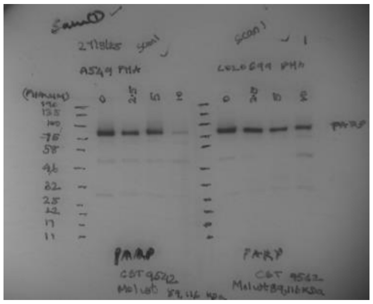
A549 PHA
COLO699 PHA
PARP
PARP

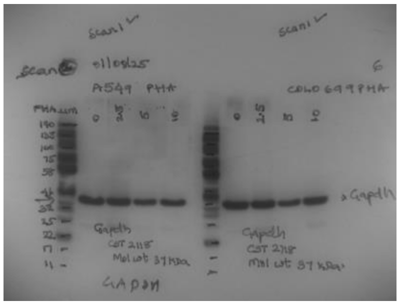
A549 PHA
COLO 699 PHA
Gapdh
Gapdh
CST 2118
Mol wt 37 kDa
GAPDH

Figure 4A

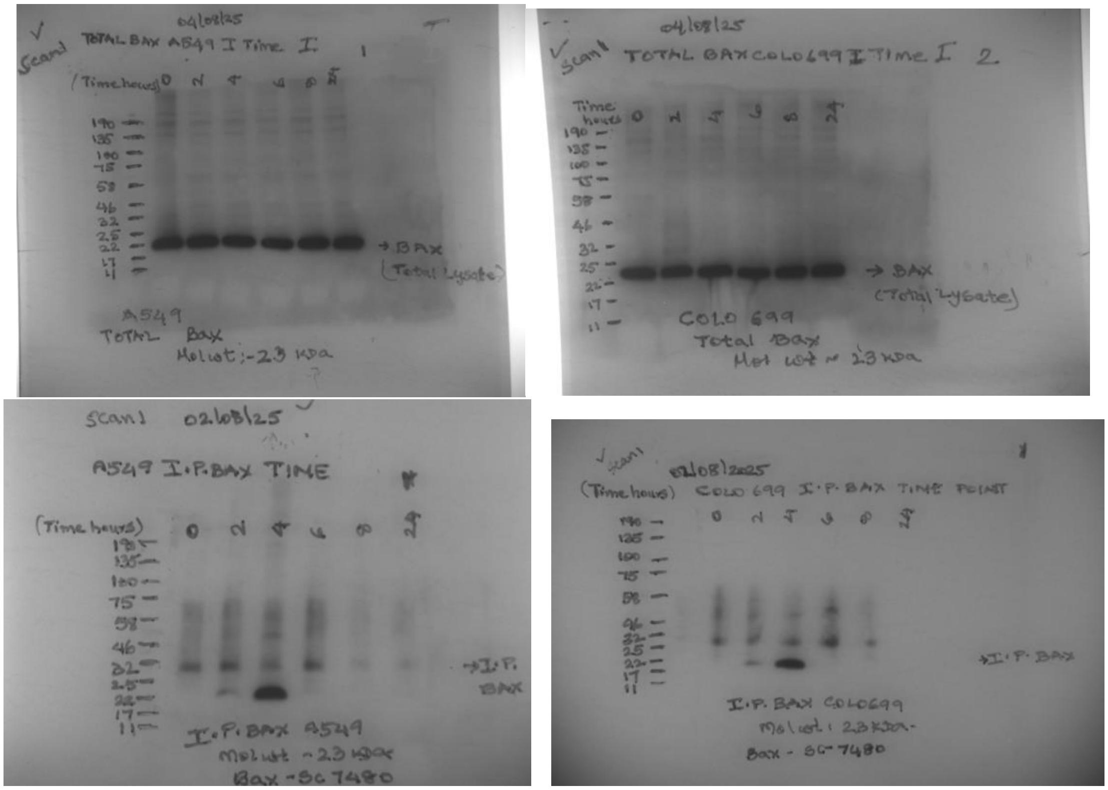

Figure 4C

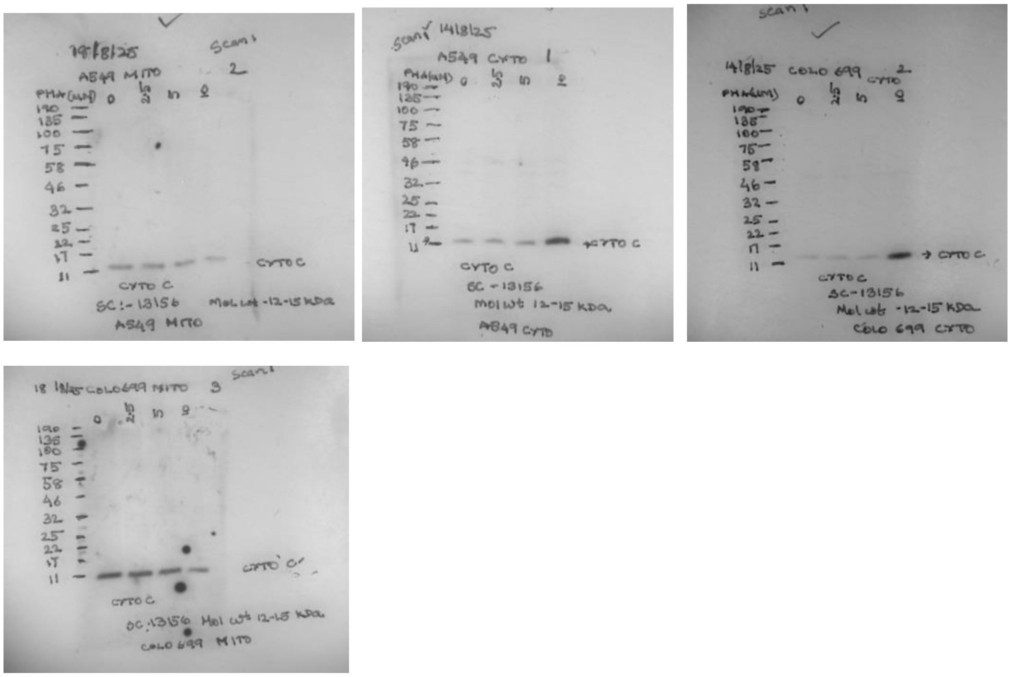

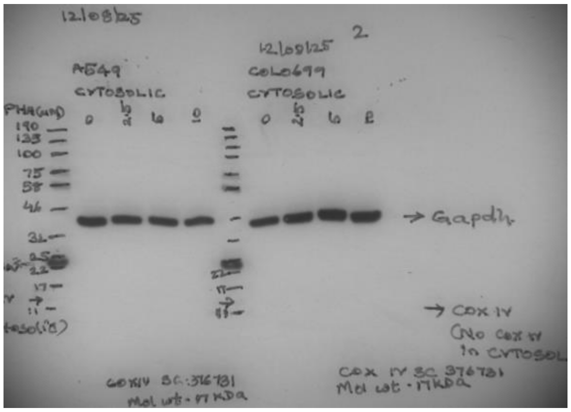
12/08/25
A549
CYTOSOLIC
12/08/25
COLO699
CYTOSOLIC
→ Gapdh
→ COX IV
COX IV SC 376781
Mol wt - 17 KDa

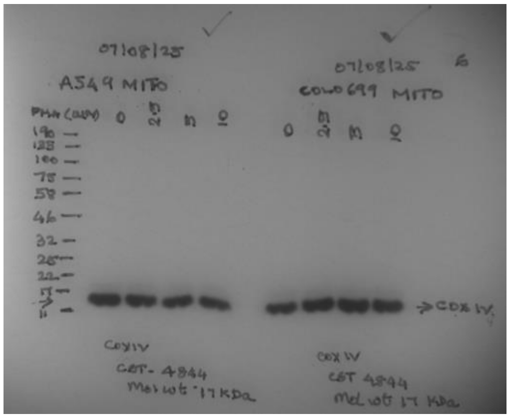
07/08/25
A549 MITO
07/08/25
COLO699 MITO
→ COX IV
COX IV
CST 4844
Mol wt 17 KDa

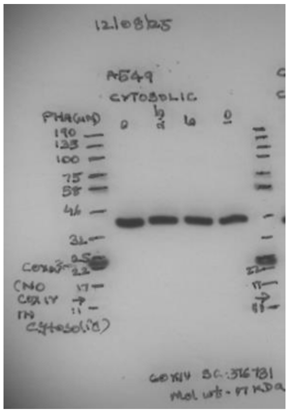
12/08/25
A549
CYTOSOLIC
COX IV SC 376781
Mol wt - 17 KDa

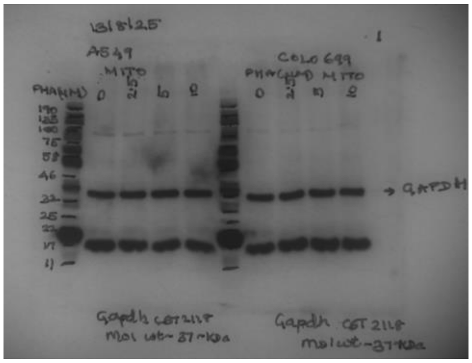
13/8/25
A549
MITO
COLO 699
MITO
→ GAPDH
Gapdh CST 2118
Mol wt - 37 KDa
Gapdh CST 2118
Mol wt - 37 KDa

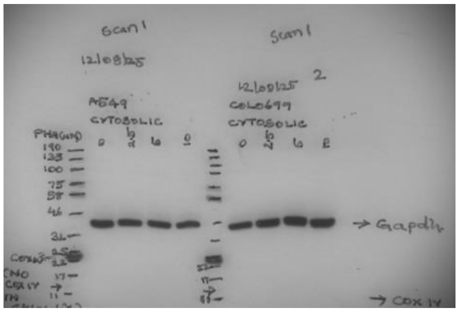
Scan 1
Scan 1
12/08/25
A549
CYTOSOLIC
12/08/25
COLO699
CYTOSOLIC
→ Gapdh
→ COX IV

Figure 6D

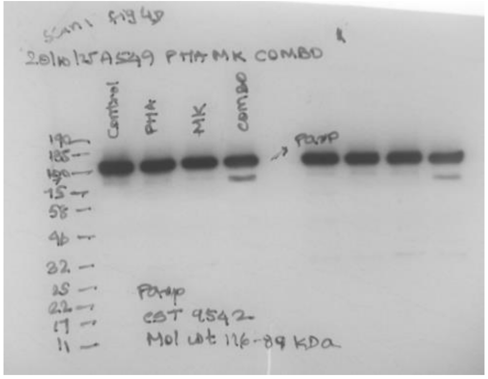
Scan 1 fig 4D
20/10/25 A549 PHA MK COMBO
Control
PHA
MK
COMBO
Parp
Parp
CST 9542
Mol wt 116-89 KDa

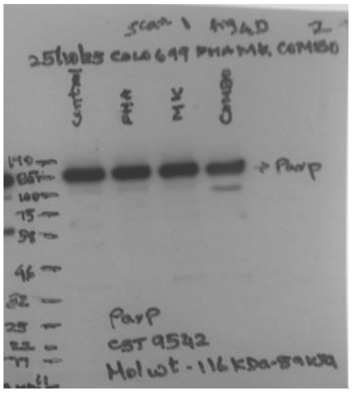
Scan 1 fig 4D
25/10/25 COLO 699 PHA MK COMBO
Control
PHA
MK
Combo
Parp
Parp
CST 9542
Mol wt - 116 KDa - 89 KDa

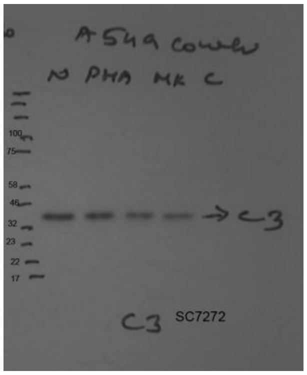
A549 Combo
N PHA MK C
→ C3
C3
SC7272

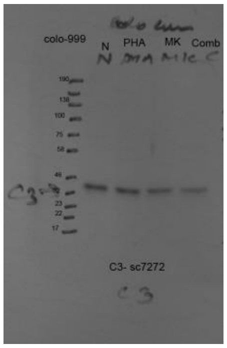
colo-999
N
PHA
MK
Comb
C3- sc7272
C3

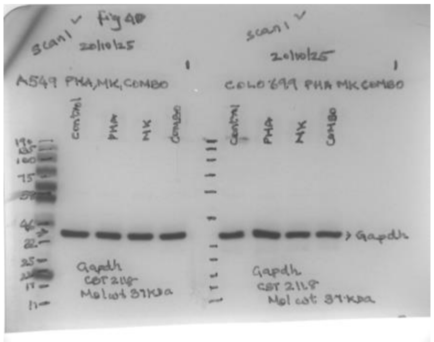
scan1
fig 4B
scan1
A549 PHA, MK, COMBO
COLO 699 PHA MK COMBO
Control
PHA
MK
COMBO
Gapdh
Gapdh
CST 2118
Mol wt 37KDa
Gapdh
CST 2118
Mol wt 37KDa

Figure S2.

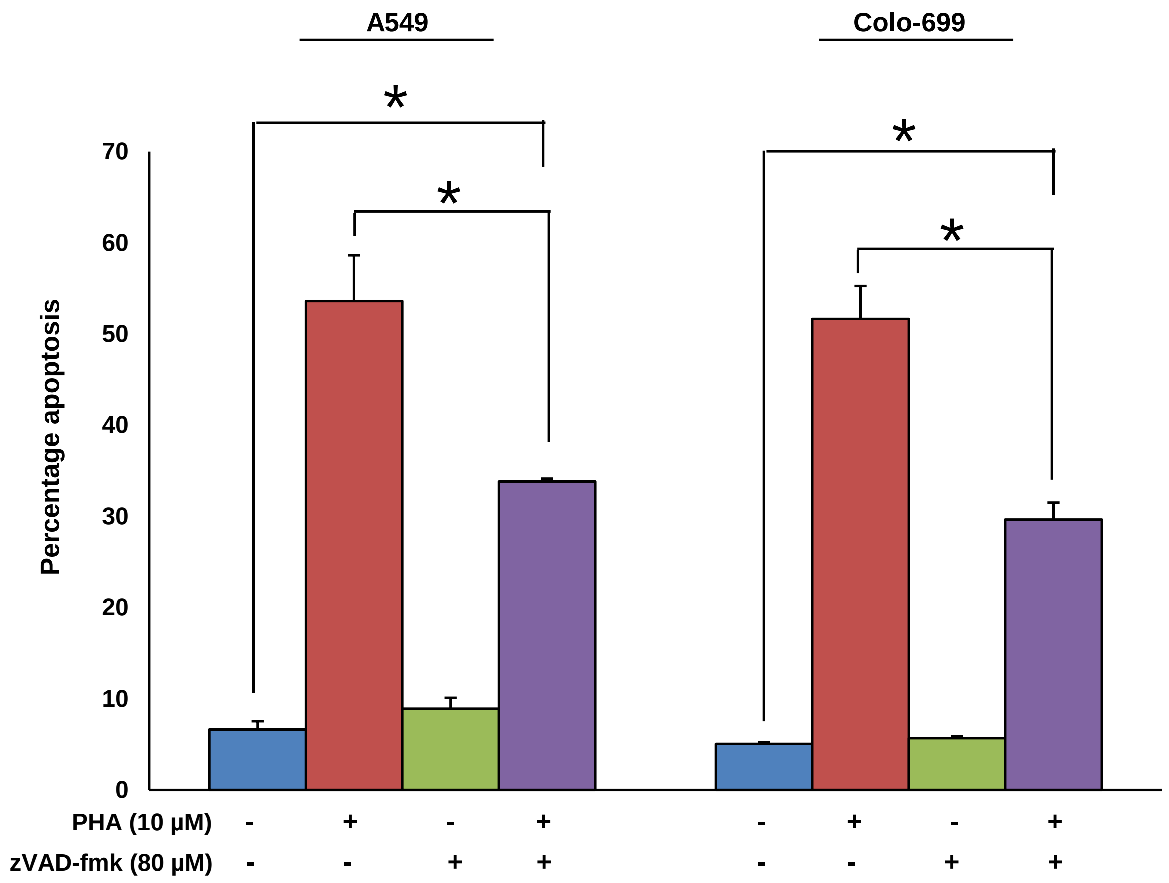

Figure S2. Effect of universal caspase inhibitor; z-VAD/fmk on PHA induced apoptosis in NSCL cells. A549 and Colo-699 cells were pretreated with 80 µM z-VAD/fmk for 3 hours and subsequently cotreated with PHA for 48 hours and cells were stained with flourescein-conjugated annexin-V and propidium iodide (PI) and analyzed by flow cytometry. Data presented in bar graphs are the mean ± SD of three independent experiments. *p<0.05

Figure S3.

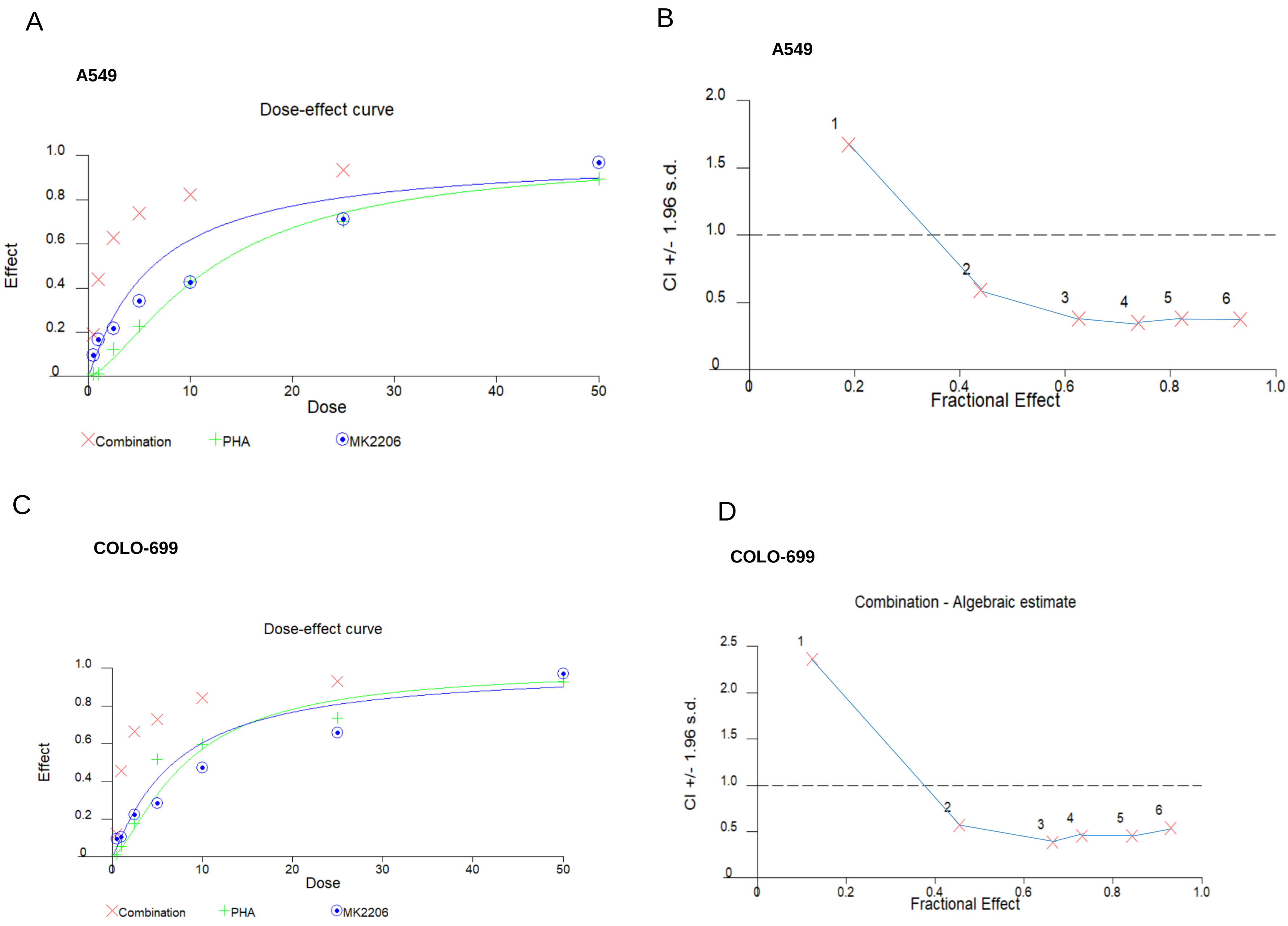

Figure S3. Synergistic interaction between c-MET and AKT inhibitors in NSCLC cells. (A,C) Dose–effect curves generated for the combination treatment of the c-MET inhibitor PHA665752 (PHA) and the AKT inhibitor MK2206 in NSCLC cell lines. The curves show the effects of single-agent and combined treatments on cell viability across increasing drug concentrations. (B,D) Combination index (CI) plots derived from dose–response analyses demonstrating the interaction between PHA and MK2206 at different fractional effects (Fa). CI values < 1 indicate synergistic interactions between the two agents. Synergistic effects were observed across a broad range of fractional effects, particularly at moderate to high inhibitory concentrations. Data were analyzed using CompuSyn software based on the Chou–Talalay method for drug combination analysis.

Figure S4.

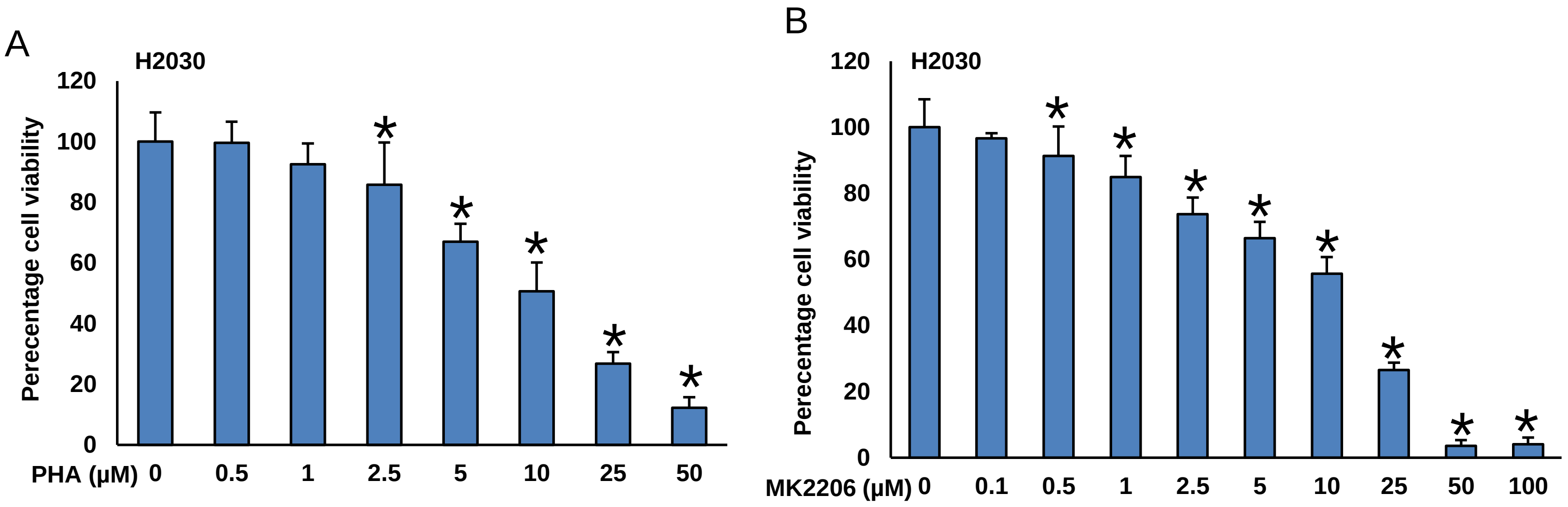

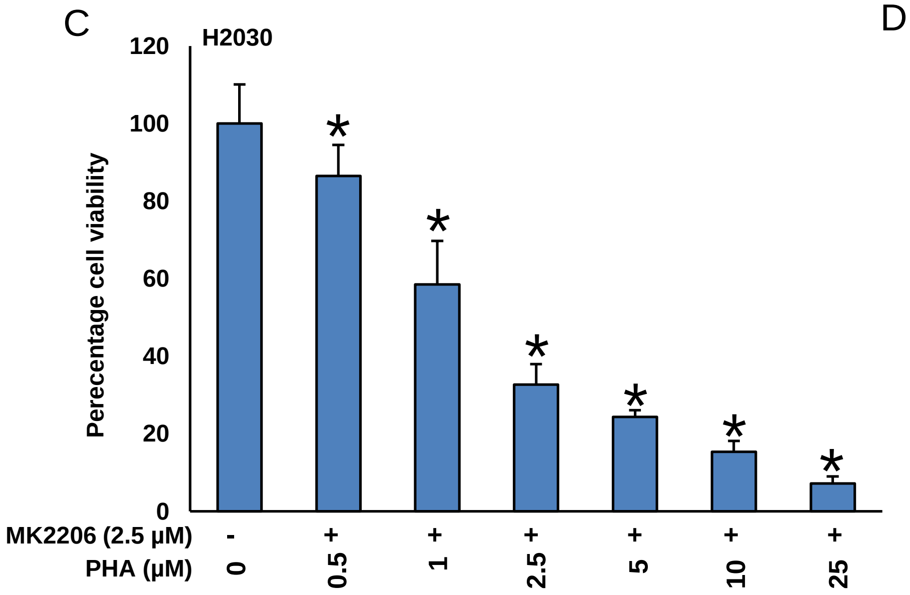

D H2030

Combination index (CI) for experimental values			
**PHA (μM)**	**MK2206 (μM)**	**Fa**	**CI**
0.5	2.5	0.1351	2.418
1	2.5	0.4144	0.672
**2.5**	**2.5**	0.6728	0.347
5	2.5	0.7563	0.354
10	2.5	0.8463	0.369
25	2.5	0.9281	0.434

Figure S4. Combined inhibition of c-MET and AKT demonstrates synergistic antiproliferative effects in H2030 NSCLC cells. (A) Effect of PHA on cell viability in H2030 cells. Cells were treated with the indicated concentrations of PHA for 48 h, and cell viability was determined by MTT assay. (B) Effect of MK2206 on cell viability in H2030 cells. Cells were treated with the indicated concentrations of MK2206 for 48 h, and cell viability was assessed by MTT assay. (C) Combined treatment with PHA and MK2206 enhances growth inhibition in H2030 cells. Cells were treated with increasing concentrations of PHA in the presence or absence of a fixed concentration of MK2206 (2.5 μM) for 48 h, followed by MTT analysis. (D) Combination index (CI) analysis of PHA and MK2206 in H2030 cells. CI values were calculated using the Chou–Talalay method with CalcuSyn software. The combination of PHA (2.5 μM) and MK2206 (2.5 μM) exhibited strong synergism (CI = 0.347). CI < 1 indicates synergism, CI = 1 indicates an additive effect, and CI > 1 indicates antagonism. Data are presented as mean ± SD of three independent experiments. *p < 0.05 compared with untreated control cells.
